# Development and validation of a semi-automated algorithm to analyze shear wave elastography clips in muscle tissue

**DOI:** 10.1038/s41598-025-05154-2

**Published:** 2025-06-20

**Authors:** Andreas Haueise, Gabriela F. Carvalho, Mehrin Azan, Dominic Gehring, Katrin Skerl, Angela V. Dieterich

**Affiliations:** 1 Department of Sport and Sport Science, University of Freiburg, Sandfangweg 4, 79102 Freiburg im Breisgau, Germany; 2 Faculty of Health, Medical and Life Sciences, Furtwangen University, Furtwangen, Germany

**Keywords:** Shear wave elastography, Muscle tissue, Semi-automated algorithm, Validation, Agreement analysis, Data processing, Image processing

## Abstract

Ultrasound shear wave elastography (SWE) is broadly used to quantify muscle stiffness. Currently, most stiffness measures are retrieved from manually placed small measurement zones, which is an operator-dependent and laborious procedure of questionable reliability. Automated time-series measurements over the full visible muscle are expected to improve measurement validity, robustness, and efficiency in larger studies. This study aimed to develop and validate a semi-automated algorithm for analyzing SWE clips of muscle tissue using the single-image, manufacturer-provided manual measurements in every image of the corresponding clips as reference. SWE clips of the relaxed and activated upper trapezius muscle of 52 healthy participants were analyzed manually and with the algorithm for the muscle’s Young’s modulus (kPa) and shear wave velocity (SWV). Results demonstrated excellent correlation between manual and algorithm measurements, Spearman’s *ρ* > 0.99, *p* < 0.001. Bland–Altman analyses indicated good method agreement with proportional biases of + 0.747 kPa and − 0.068 m/s for Young’s modulus and SWV, respectively, and widths of the limits of agreement of 8.653 kPa and 0.500 m/s, respectively. The proportional bias is within the minimal detectable change and therefore clinically negligible. These results support the algorithm as a tool enabling valid SWE time-series measurements in muscle tissue and an improved workflow.

**Supplementary Information:**

The online version contains supplementary material available at 10.1038/s41598-025-05154-2.

## Introduction

Ultrasound shear wave elastography (SWE) is a fairly novel imaging technique aiming to quantify mechanical tissue properties closely linked to the concept of stiffness^1^. SWE is based on B-mode ultrasound and additionally uses focused sound beams, so called “push beams”, causing minimal displacement within the tissue and therewith the propagation of shear waves^1^. The ultrasound system measures the propagation speed of these shear waves (shear wave velocity, SWV), and calculates the Young’s modulus as a mechanical measure of tissue stiffness in units of Pascal (typically kPa) based on the SWV^1–3^. Greyscale ultrasound images of the tissue with a superimposed heatmap (elastogram), visualizing the distribution of stiffness are generated and can be quantitatively analyzed^2^. This technique was developed for diagnostic purposes to identify fibrous or cancerous regions in mainly homogeneous (isotropic) tissue like liver or breast^1^. In recent years, SWE was validated in laboratory settings using phantoms or ex vivo animal muscle to assess its applicability on non-uniform (anisotropic) tissues like skeletal muscle^2,4^. Subsequently, the validity was demonstrated in vivo in human participants^3,4^ and studies using SWE in muscle tissue are increasing^5,6^. Nowadays, SWE is widely used in muscle tissue to assess muscles’ biomechanical properties in various contexts such as diagnostics, pain research or interventional studies^5–7^. However, several methodological problems have been identified in these contexts, such as insufficient reporting of technical specifications and non-standardized image analyses^5,6,8^.

The standard procedure of image analysis of SWE data is placing measurement zones or regions of interest (ROI) using the manufacturer-provided software^9^. Since SWE was developed for diagnostic purposes, most software enables the manual placement of circular measurement zones within single ultrasound frames to measure the stiffness of distinct, potentially malignant regions of high stiffness^10,11^. In cancer or fibrosis diagnostics, clinicians are encouraged to find the best frame within the imaging sequence, and to place the measurement zone where they expect pathologic findings^11^. In muscle tissue, stiffness is typically distributed inhomogeneously^5,6,9^. Placing measurement zones manually may be problematic, as they may be placed where the tissue properties appear representative according to preconceived assumptions and hypotheses^6^. Additionally, previous studies have suggested that small measurement zones may not adequately represent the entire muscle, and that larger zones could help address this limitation^6,12–15^. Further potential bias might be introduced by measuring stiffness in single images^6^, since muscle stiffness is not only unevenly distributed spatially but also temporally^9^. The distribution of stiffness in ultrafast SWE scanning appears to be rapidly fluctuating^9^, and is influenced by movement or compression artefacts^8,16^. Therefore, using single images may not provide generalizable stiffness measures^6^.

Computer-based analyses using standardized measurement zones covering as much of the muscle as technically possible, could overcome these limitations by enhancing the representativeness of measurements for the whole muscle and reducing potential bias introduced by the rater’s expectations^6^. Since raw numeric data is often unavailable without distinct research interfaces, which to our knowledge are either discontinued or were never provided by some manufacturers, custom-made image-based analysis is required. Some studies have already successfully approached data analysis using custom-made analysis tools^17–29^ and at least one open-source program for this purpose exists^30^. To our knowledge, statistical validation measures have not been provided. The use of computed image analysis is an advancement over the manual placement of measurement zones, yet it is mostly used in single ultrasound frames. The analysis of clips (time-series) would provide more robust and representative measures of muscle stiffness^6^, accounting for the variability of stiffness and reducing potential influences of artefacts^6,8,9,16^.

This study aimed to develop a software tool based on a published SWE algorithm^10^ that allows users without programming knowledge to perform computed SWE clip analyses. The previously published SWE analysis algorithm was developed for breast tissue which is an isotropic tissue whereas muscle tissue is anisotropic. Thus, the second aim of this work was to assess the convergent construct validity and agreement of the newly developed software tool adapted for muscle tissue with the manufacturer-provided analysis software. This newly developed tool could target the previously stated limitations in current analysis methods of SWE data. Further, the new approach would facilitate the standardization and enhance the robustness of SWE data analysis in muscle tissue.

## Methods

### Algorithm and graphical user interface (GUI)

**Fig. 1:**
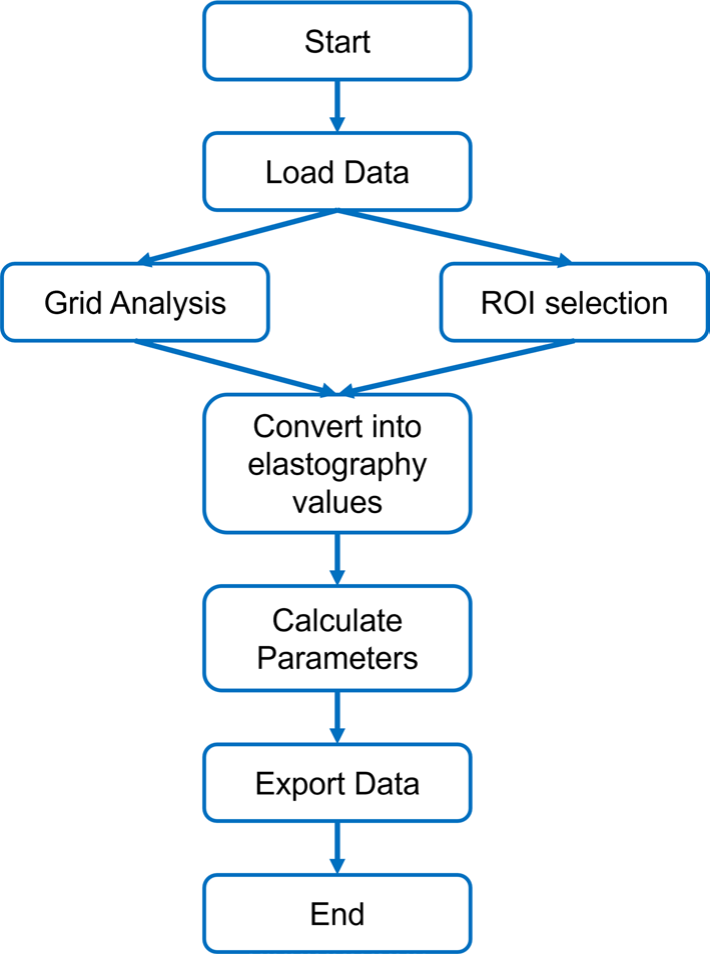
Flowchart showing the general working principle of the developed algorithm. The user can choose whether a grid or a hand drawn region of interest (ROI) is analyzed.

The software tool being developed is based on the algorithm developed and described by Skerl et al.^10^, and was adapted to analyze SWE clips in muscle tissue using MATLAB (Version 2021b, The MathWorks Inc., Natick, USA). The tool processes DICOM-format clips from Aixplorer systems that display both the b-mode image and the greyscale image containing the superimposed elastogram in a vertical arrangement, as these are the most commonly used settings. DICOM is the most common standard for image extraction from the system as it contains more meta-data in addition to the video and image. The vertical image orientation can display the full width of most transducers compared to the side-by-side orientation, enabling users to freely position the elastogram within the full visible muscle section. Further, the algorithm was developed to process elastograms displayed with 50% opacity, since this is the default setting in Aixplorer devices and yielded the best results in the development of the original algorithm^10^. The workflow of the algorithm is shown in Fig. 1. Following data selection and loading, the SWE image is segmented automatically based on the known approach^10^. However, minor changes were applied to adapt to the differences in the image representation as the original approach was developed to analyze breast images. If the image is displayed differently, or the automatic segmentation is not satisfactory, users can choose to segment the image manually. The clips are processed using the b-mode sampling frequency, each frame is analyzed individually, without averaging. Size-scaling is achieved by analyzing the measurement bar displayed within the ultrasound image. Both the original and the adapted algorithms are developed for images shown in the temperature gradient style with representing low elasticity values in blue and high values in red. For instance, the SWE image is surrounded by an orange line in the exported videos whereas it is surrounded by a white line if single images are exported. In the currently available version, the user can choose which image shall be displayed and whether a grid containing 4 × 4 mm squares or a hand drawn rectangular ROI shall be analyzed. This enables the analysis of the largest possible area or multiple smaller areas, to assess spatial differences within the clip. Currently, the tool does not allow to set free-form ROIs. Per default, the ROI is defined in the first frame of the clip, but the user can choose any frame for this step. Once the ROI is selected this is consistently used for every frame of the clip. However, it is also possible to analyze distinct frames of each clip. The tool offers four ways of displaying the ultrasound image: the full frame including all information that is displayed by the device, the segmented elastogram, the preview of the grid, or the selected hand-drawn ROI. The user can again choose to display any of the frames of the clip. Using the input of the elasticity range’s maximum value, the algorithm assigns each colored pixel a numeric value corresponding to Young’s modulus in kPa. For this step it is necessary that the maximum value of the elasticity color bar is adjusted to the current maximum value and in accordance to the one set in the GUI, i.e. the elastograms should comprise a large range of colors within the spectrum instead of being mostly dark blue or bright red. Since reliable stiffness assessment depends on high-quality imaging^5,6^, and Aixplorer devices do not provide stability or shear wave tracking metrics across all presets and transducers, including the musculoskeletal preset, the software estimates image quality by reporting the percentage of pixels with elasticity values (color) within the ROI per frame.

According to the user’s selection the parameters ratio of elasticity values within image section, mean, median, standard deviation, interquartile range, maximum value, number and percentage of values exceeding the top 2% of the maximum value are automatically retrieved for each image comprised in the chosen clip and provided as a spread sheet for statistical evaluation.

**Fig. 2:**
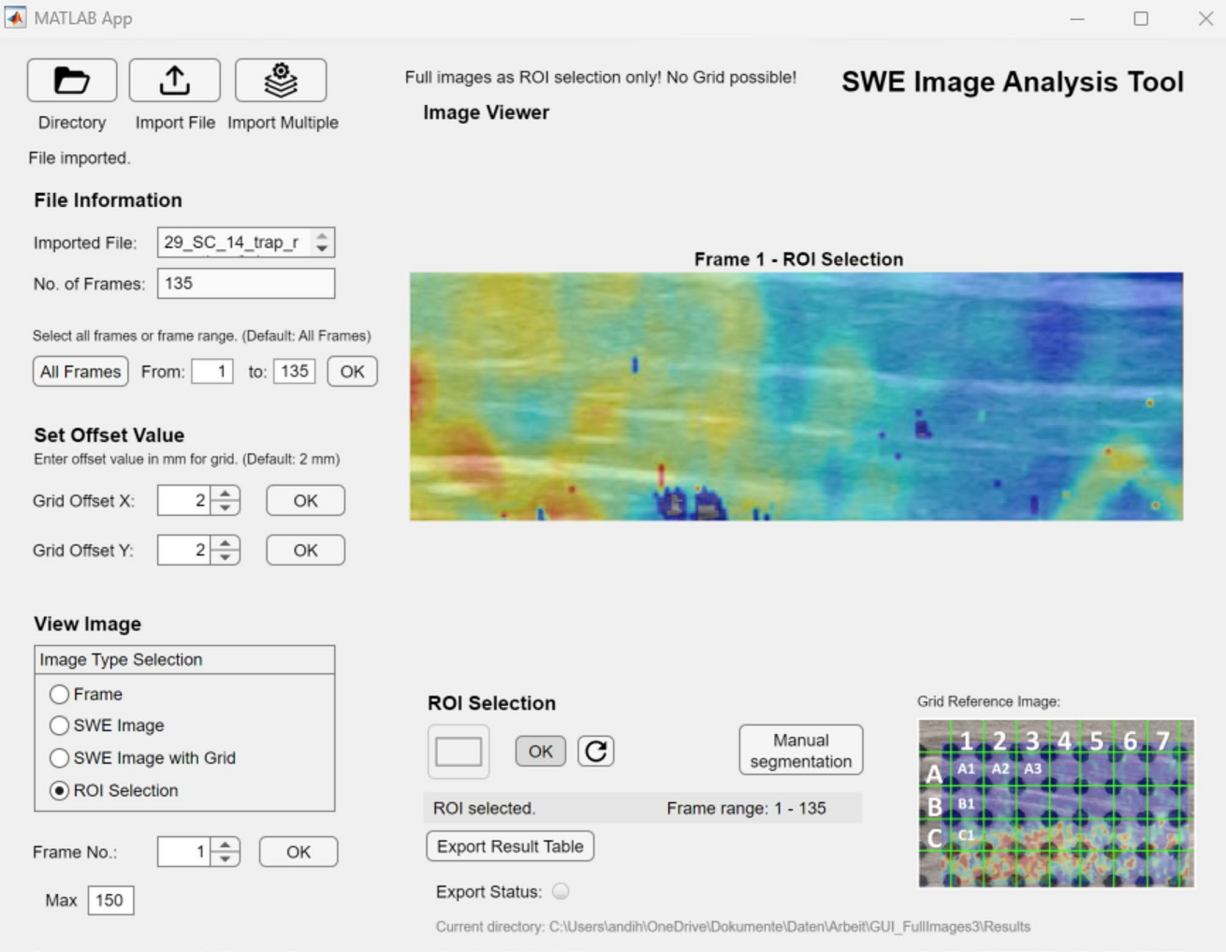
Graphical User Interface (GUI) of the algorithm used to evaluate the SWE clips.

As interface for the analysis a graphical user interface (GUI) was created with MATLAB’s App Designer program as shown in Fig. 2. The developed GUI has various functions to facilitate users to analyze SWE images without programming knowledge. Each component also has a tooltip to inform the user on what each component does. Alongside the code files, a short guide to use the GUI, can be found in the GitHub repository.

### Data collection

This validation and agreement study was conducted in accordance with the Declaration of Helsinki, with written informed consent obtained from all participants prior to data collection. The study was part of a larger project and approved by the local ethics committee of Furtwangen University (Protocol number: 24-001). Reporting of this validation and agreement study is based on the GRAS^31^ and COSMIN^32^ guidelines, as far as applicable.

SWE data was acquired in a sample of 52 participants (Table 1). Volunteers aged 18 to 60 years were included in the study, provided they had no systemic diseases or were not using medications that could potentially affect the neuromuscular system, such as muscle relaxants or antidepressants. For this validation study, data from the right-hand side upper trapezius muscle was used, since this muscle offered a large enough area to easily standardize the ROI in the manual analysis by tracing the outer border of the whole elastogram box. Data acquisition was carefully conducted with respect to the underlying principles that enable the use of SWE in anisotropic tissue, like muscle, by applying minimal pressure with the transducer and orienting the transducer perpendicular to the bodies’ surface in the longitudinal imaging plane^9^. The upper trapezius muscle was recorded with participants lying in prone position on an examination bed with their cervical spine in a neutral position. The muscle was palpated and then identified using b-mode ultrasound, starting in the transversal plane, approximately at 50% of the distance between the occipital bone and the acromion. Once the trapezius muscle was identified in the transversal plane, the transducer was rotated to the longitudinal plane and positioned to find traceable muscle fibers and to present them horizontally in the middle of the image. The system mode was switched to shear wave elastography and the elastogram was set to maximum width (3 cm) and to a height large enough to cover the full muscle section. Once the muscle was displayed correctly, two repetitions of 10 s of resting state and muscle activation (lifting the head up from the examination bed) were recorded. These conditions were chosen to assess both conditions relevant for using SWE in research and clinical applications. SWE data was acquired using an ‘Aixplorer Ultimate V12’ SWE system with a ‘SL15-4’ linear transducer (V12.3.1.849, SuperSonic Imagine, Aix-en-Provence, France). SWE data was recorded as clips of 10 s, with B-mode and SWE frame rates of 11 Hz and 1.6–2.0 Hz, respectively. The musculoskeletal preset was used, with a standard mode between resolution and penetration. The elasticity range was set to the device’s maximum range of up to 300 kPa for data collection. Before exporting the data as DICOM files for analysis, an individually chosen range displaying a large range of colors was set to enable a high resolution of the colormap. Pilot tests demonstrated that a high resolution yielded the best agreement between the algorithm’s and the Q-Box’s results. The maximum value of the elasticity range, was used as the input in the software tool accordingly. Aixplorer’s default colormap using the temperature gradient was used.

**Table 1:** Sociodemographic data of the study population, mean ± SD.

Number of participants	52
Gender	
Female	37
Male	15
Age (in years)	29.7 ± 10.7
Height (in cm, mean ± SD)	171.2 ± 7.7
Weight (in kg, mean ± SD)	67.1 ± 10.7

### Data processing

In order to prepare the data for the validation analysis, all clips were processed in two ways: first, the ‘manual analysis’ using the system-provided software ‘Q-Box’ as the reference standard, and second, the ‘semi-automated analysis’ using the newly developed GUI. Since ‘Q-Box’ does neither allow the analysis of the full elastogram nor of clips, the assessor analyzed every distinct elastogram of every clip by tracing the outer border of the whole elastogram box with the ‘manually circumscribe’ option. In this way the complete visible area of the trapezius muscle was analyzed. Mean Young’s modulus and SWV including the standard deviations displayed by the software were extracted and stored in a distinct spreadsheet for each clip, enabling the statistical analysis of the full time-series, although this option is not natively provided by the manufacturer’s software.

Afterwards, all clips were processed using the GUI by placing a rectangular shape closely matching the area used in the manual analysis. As previously described, the elasticity range was chosen for each clip individually to achieve a large distinctly-color-coded range of elasticity values. Within the GUI, the maximum elasticity value was set corresponding to the elasticity range of each clip. Both the system-provided software and the algorithm express stiffness as Young’s modulus in kPa, suitable for interpreting isotropic tissues like breast^1^, for which the algorithm was developed^10^. For accurate interpretation in anisotropic muscle tissue, conversion to shear modulus or the reporting of SWV is necessary^2^. To target this, the algorithm’s results were recalculated to SWV based on the established formulae^2,3^. These underlying equations are in parts based on approximations, potentially introducing inaccuracies^8^. Therefore, we decided to only use Young’s modulus and SWV for further analyses, and to not calculate shear modulus. The most recent recommendations for the reporting of SWE measurements in muscle tissue state that only SWV should be used^5,6,8^. Lastly, the mean Young’s modulus and SWV of all elastograms in a clip was calculated for both analysis methods and used for statistical evaluation.

### Statistical analysis

The statistical analyses were performed using MATLAB (Version 2023b, The MathWorks Inc., Natick, MA, USA) and jamovi (Version 2.3.28, The jamovi project, Sydney, Australia). The data was tested for normality using the Shapiro–Wilk test. Since the assumptions of a normal distribution could not be met for all variables (p < 0.05), non-parametric tests were used. Median and interquartile range (IQR) were calculated as descriptive statistics. To test for the convergent criterion validity of the algorithm, the correlation between both methods was estimated using Spearman’s rank correlation^33^. Correlation coefficients below 0.39 were considered as weak, those between 0.40 and 0.69 as moderate, 0.70–0.89 as strong, and values above 0.90 as very strong correlations^34^.

The agreement between the processing methods was assessed using the Bland–Altman analysis^35^ with subsequent hypothesis testing of agreement, as proposed by Shieh^36^. The aim of agreement analyses is to assess whether two methods agree in a way to be used interchangeably^35^. Several steps are necessary to correctly perform a Bland–Altman analysis^35,37–43^. A priori, the measures of clinical relevance were determined*.* For this study we considered that the width of the Limits of Agreement (LoA) should not exceed the minimal detectable change (MDC) of SWE measurements, indicating that the potential bias lies within the measurement error and is therefore negligible^44^. Previous research states that the MDC for SWE measurements of the upper trapezius muscle ranges between 10.23–27.90 kPa and 1.85–3.04 m/s in Young’s modulus and SWV, respectively^45,46^. Following, the differences between the two analysis methods were calculated, a scattergram of differences on averages was created containing a superimposed a horizontal line, representing the mean difference (bias)^35^. If zero was present within the 95% confidence interval (CI) of the bias, it was considered non-significant^41^. Third, the upper and lower LoA were calculated by using ± 1.96 × the standard deviation of the differences and also visualized using horizontal lines^35^. Once the final Bland–Altman plot was completed, the scattergram was visually inspected regarding trends within the data^37–43^. During visual inspection trends indicating underestimation at the lower end of measurement values and overestimation at the upper end of measurement values were present and were assessed statistically using the line of best fit for linear regressions^37–39,41–43^. Statistically significant regression models with slopes differing from zero indicate either the presence of a proportional bias, i.e. that the bias changes with the magnitude of the measurements or could be influenced by non-normally distributed differences^37,38,41–43^. To account for linear trends Bland & Altman recommend to (a) assess approximate normality of the differences using histograms (Supplementary Fig. S1 and S2) and (b) to logarithmically transform the data and repeat all aforementioned steps^37,38,42,43^. If the linear trends persist after log-transformation of the data, this indicates the presence of a true proportional bias, if not the apparent trends occur due to non-normality of the data and can be neglected^42^. Based on these recommendations, all steps of the Bland–Altman analysis were conducted using the log-transformed and the non-transformed data.

Additionally, Shieh’s agreement hypothesis test was conducted using the *SimplyAgree* package for jamovi^47^. This test compliments the Bland–Altman analysis by offering an objective statistical measure to assess whether methods agree in a way that allows them to be used interchangeably or not. Shieh’s test was conducted using both datasets, the log-transformed and the non-transformed data. *SimplyAgree* provides the user with the 90% CI and a statement regarding the rejection of the null hypothesis^47^.

## Results

**Fig. 3:**
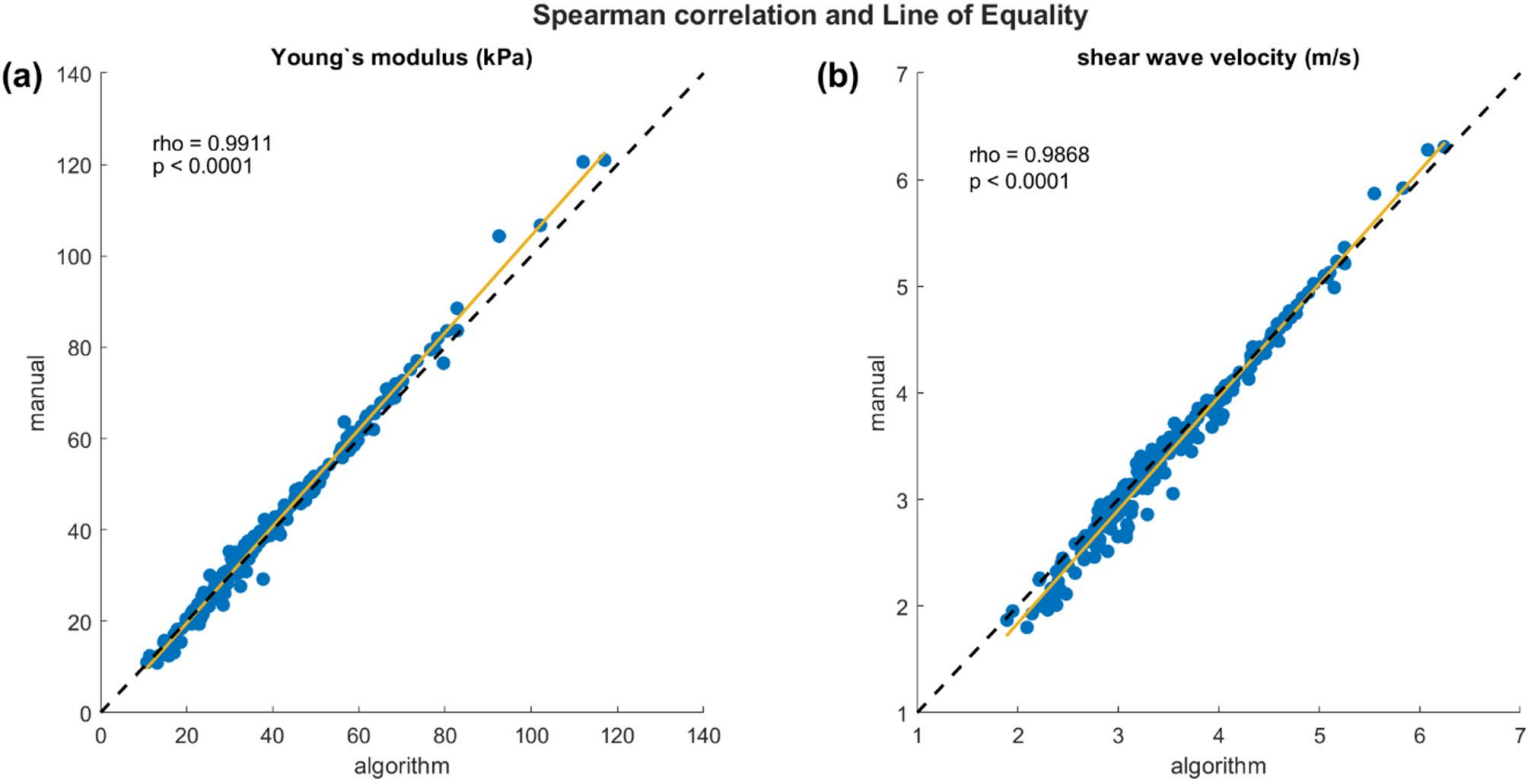
Spearman correlation plots with Line of Equality (black dashed line) and line of best fit (yellow line), expressed as (**a**) Young’s modulus and (**b**) shear wave velocity. The correlation is statistically significant and considered very strong (*ρ* > 0.98, *p* < 0.001) in both measurement units.

From a total of 208 available clips, n = 206 were included in the analysis, n = 2 files were technically damaged and not readable. Table 2 shows the descriptive statistics of both analysis methods. The convergent construct validity analysis using Spearman’s correlation coefficient (*ρ*) revealed very strong positive correlations between the manual and the algorithm-based analysis in both measurement units (Young’s modulus: *ρ* = 0.991, *p* < 0.001; SWV: *ρ* = 0.987, *p* < 0.001). Figure 3 displays the correlation plots with a line of equality.

**Table 2:** Descriptive statistics of both analysis methods, expressed as Young’s modulus in Kilopascal (kPa) and shear wave velocity (SWV) in m/s, median and interquartile range (IQR).

	Included clips n=	Young’s modulus (kPa) median (IQR)	SWV (m/s) median (IQR)
Algorithm	206	33.79 (23.76)	3.36 (1.13)
Manual	206	34.59 (25.13)	3.32 (1.16)

Assessing the agreement between both methods using the Bland–Altman analysis for the dataset for Young’s modulus revealed a mean difference (bias) of + 0.747 kPa with 95% CI ranging from 0.44 to 1.05 kPa, not including zero and therefore considered significant. 95.6% of measurements are within the LoA. The LoA had a width of 8.653 kPa (ranging from − 3.579 kPa to 5.074 kPa). This was confirmed by the Shieh’s agreement hypothesis test (90% CI ranging from − 3.86 to 5.35, *p* > 0.05), indicating that the null hypothesis could not be rejected and therefore the algorithm’s analyses cannot be used interchangeably with Q-Box values when using Young’s modulus. The assessment of the visual trends within the scattergram using a linear regression revealed a statistically significant model with slope and intercept deviating from zero (F (1, 204) = 92.5, *p* < 0.001, slope = 0.063, intercept = − 1.752), indicating a proportional bias or heteroscedasticity^20^. After log-transformation of the data the bias was non-significant (+ 0.008, 95% CI ranging from − 0.002 to 0.017), while the linear regression still revealed a statistically significant model (F (1, 204) = 43.1, *p* < 0.001, slope = 0.058, intercept = − 0.201), indicating a true proportional bias. Shieh’s test revealed that the null hypothesis could be rejected after log-transformation (90% CI − 0.13 to 0.15, *p* < 0.05), indicating that the two methods can be used interchangeably, when log-transforming Young’s modulus values.

**Fig. 4:**
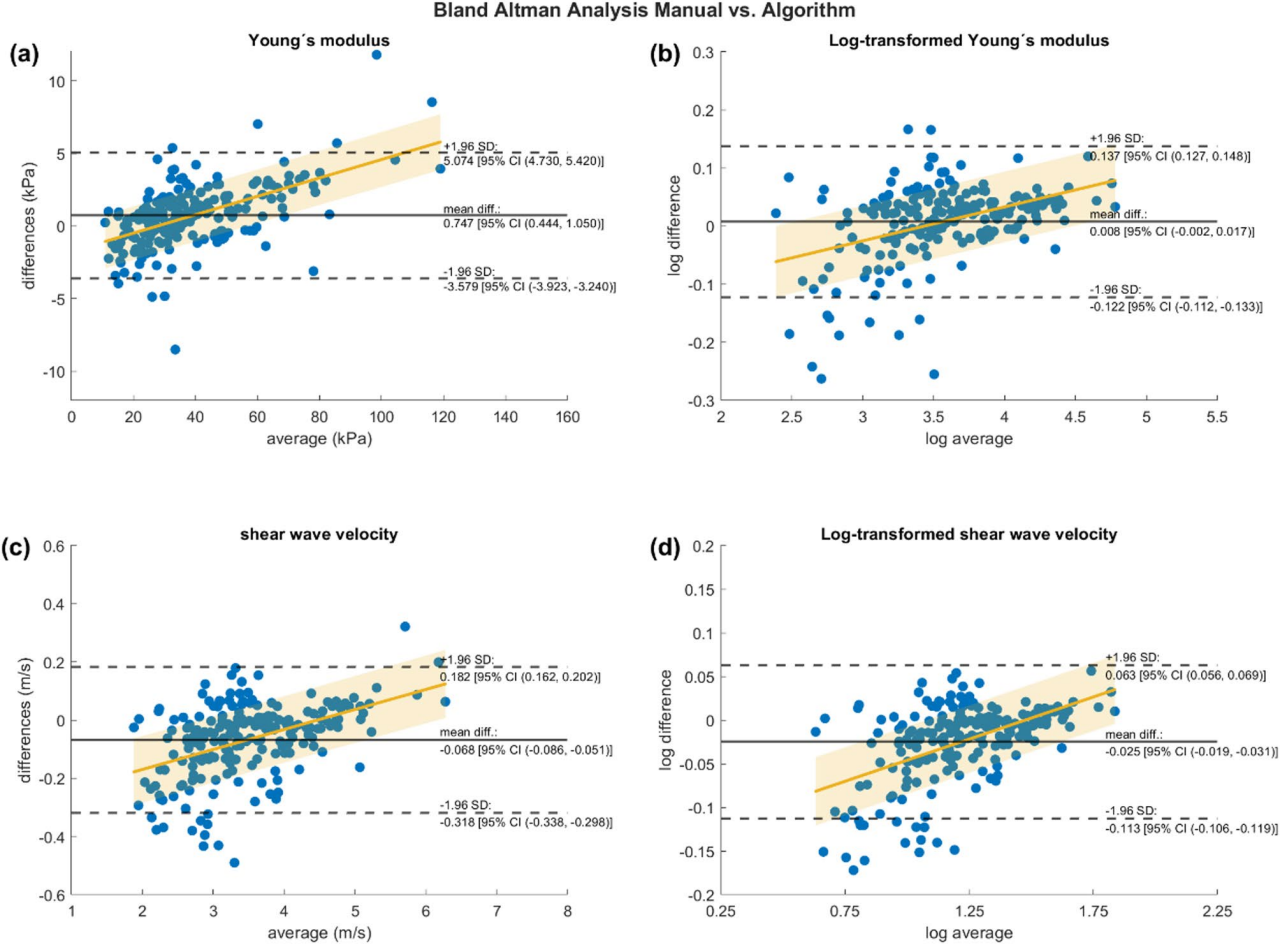
Bland–Altman plots with superimposed horizontal lines for the bias (solid black line) and limits of agreement (dashed lines), and the line of best fit of the linear regression with its 95% confidence intervals (yellow line and shaded area). Analyses are presented as: (**a**) Young’s modulus, (**b**) log-transformed data based on Young’s modulus, (**c**) shear wave velocity, and (**d**) log-transformed data based on shear wave velocity. The plots indicate that the bias is close to zero, with most values within the limits of agreement across all conditions, although visual linear trends are present. The limits of agreement span 8.653 kPa for Young’s modulus and 0.500 m/s for shear wave velocity. The predefined measure of clinical relevance specifies that the width of the limits of agreement should not exceed the minimal detectable change of shear wave elastography, which ranges from 10.23–27.90 kPa and 1.85–3.04 m/s for Young’s modulus and shear wave velocity^45,46^, respectively.

For the analysis using SWV the bias was − 0.068 m/s with 95% CI ranging from − 0.085 to − 0.051 m/s, not including zero and therefore considered significant. 93.7% of measurements are within the LoA. The width of the LoA was 0.500 m/s (ranging from − 0.318 m/s to 0.182 m/s). Shieh’s agreement hypothesis test (90% CI ranging from − 0.33 to 0.19, *p* < 0.05) indicated that the null hypothesis could be rejected and therefore the algorithm’s analyses can be used interchangeably with Q-Box values when using SWV. The assessment of the visual trends within the scattergram using a linear regression revealed a statistically significant model with slope and intercept deviating from zero (F (1, 204) = 54, *p* < 0.001, slope = 0.069, intercept = − 0.307). After log-transformation of the data the bias was still significant (− 0.025, 95% CI ranging from − 0.031 to − 0.019). The linear model approached but did not reach statistical significance, while slope and intercept still deviated from zero, indicating a true proportional bias (F (1, 204) = 75.2, *p* = 0.059, slope = 0.096, intercept = − 0.1426). Shieh’s test revealed that the null hypothesis could be rejected after log-transformation (90% CI − 0.12 to 0.07, p < 0.05), indicating that the two methods can be used interchangeably, when log-transforming SWV values. Figure 4 displays the visual Bland–Altman plots including horizontal lines representing the bias and the LoA as well as the lines of best fit of the linear regressions in yellow in (a) Young’s modulus, (b) log-transformed Young’s modulus, (c) SWV, and (d) log-transformed SWV.

## Discussion

This study a) successfully developed the proposed software tool and b) shows that the results the algorithm provides are valid and largely agree with the current standard. The implications of statistical measures, clinical interpretation of the results and limitations of this work are discussed in the following.

Based on the very strong positive correlations between the manufacturer-provided manual analysis and the semi-automated analysis, the algorithm provides valid stiffness measures from SWE clips in muscle tissue^33,34^. This allows analysis of clips that often comprise hundreds of images in an instance overcoming the cumbersome time-consuming manual evaluation. The correlation coefficients were almost equal for both measurement units, indicating that the convergent criterion validity is given for analyses using Young’s modulus and SWV^33^. This conclusion is valid under the assumption that the system-provided software detects ‘true’ stiffness measures. As previously stated, SWE has been validated for the use in muscle tissue under several conditions in vitro and in vivo, using ‘SuperSonic Imagine’s’ devices^2–4^. Therefore, we assume that the manual analysis is a trustworthy reference for the here presented validation study.

The results of the Bland–Altman analysis, along with the findings from Shieh’s agreement hypothesis test, largely indicate a generally good level of agreement between the two measurement methods. However, several aspects should be discussed in detail. First, in both measurement units a bias was demonstrated^41^. Compared to absolute values, the biases are small and close to zero, indicating good agreement^39^. Despite the observed biases, about 95% of values fell within the LoA, indicating that the main assumptions of the Bland–Altman analysis were met^35^, and the majority of values are within the acceptable range. Visual inspection of the data revealed linear trends within the differences, which were statistically confirmed by significant linear regression models. Since the trends persisted even after log-transformation, the present biases need to be viewed as proportional, meaning differences change proportionally with the magnitude of measured values^42^. These trends within the data indicate that the algorithm is best suited to distinguish colors when the resolution of the colormap is higher, i.e. the maximum elasticity value is adjusted to provide a large range of colors to be displayed. This highlights the importance of exporting elastograms in elasticity ranges that allow for a large range of colors to be displayed, rather than color-uniform elastograms. Shieh’s agreement hypothesis test revealed that the algorithm’s analyses are interchangeable with Q-Box values when using SWV or logarithmic scaling (*p* < 0.05), but not when using Young’s modulus (*p* > 0.05)^36^.

Although the significant proportional bias systematically influences the obtained data, the results also need to be interpreted from a point of clinical relevance. As previously stated, we chose the MDC of SWE measurements as the measure of clinical relevance, against which we compared the width of the LoA. This approach enables an assessment of whether the detected bias falls within the margin of measurement error and can therefore be considered negligible, as changes of this magnitude would not be reliably measurable^44^. The MDC of SWE in muscle tissue was assessed in several studies before^45,46,48–50^. Xie et al. (2019) as well as Kozinc & Šarabon (2020) calculated the MDC of the measurements within reliability studies using SWE for muscles of the cervical and axioscapular regions and of the upper trapezius muscle specifically^45,46^. Both measurement set-ups differed from the present study’s data acquisition, as participants were recorded in a seated position^45,46^ and in the study of Kozinc & Šarabon also with varying shoulder abduction angles ranging from 0° to 60°^45^. Appropriately for muscle tissue, both studies reported measurements as shear modulus in kPa^2^, therefore recalculation into Young’s modulus and SWV was necessary for comparison. Xie et al. reported MDC values for the anterior upper trapezius and posterior upper trapezius separately, while the posterior measurement position is closest to the position used in the present data collection^46^. In the posterior position and dependent on the number of measurement repetitions, the MDC ranged between 3.41 kPa and 3.99 kPa shear modulus^46^, representing approximately 10.23 kPa to 11.97 kPa Young’s modulus or 1.85 m/s to 1.99 m/s SWV. In the 0° condition of Konzinc & Šarabon’s study, which is most comparable to the present data, the MDC was 9.3 kPa shear modulus^45^, representing approximately 27.9 kPa or 3.04 m/s in Young’s modulus and SWV, respectively. The width of the LoA in the present study is 8.653 kPa Young’s modulus or 0.500 m/s SWV. Therefore, the MDC of the trapezius muscle, as reported by Kozinc & Šarabon, is more than three times the width of the LoA in Young’s modulus and more than six times in SWV^45^. Although clearly lower, the MDC reported by Xie et al. is still more than double the width of the LoA in SWV and almost one standard deviation higher than the mean difference in Young’s modulus^46^. Therefore, the bias detected in the agreement analysis of the algorithm appears to be clinically not relevant and lies within the margin of measurement error of SWE itself. Hence, the algorithm can be used to facilitate and enhance scientific work using SWE in muscle tissue, by allowing a standardized, blinded and semi-computed analysis of the captured clips. When using the algorithm for research purposes, one needs to be aware of several assumptions that need to be met in order to achieve valid results. This will be discussed in the following.

Due to discrepancies in the results of the Bland–Altman analysis after the conversion from Young’s modulus to SWV, it is likely that the calculation methods used in the Aixplorer ultrasound device differ from those reported in the literature^2,3^. Aixplorer systems appear to exhibit a rounding error, evident to users as the maximum values don’t precisely match the expected results from published formulas when switching between Young’s modulus and SWV settings. Ultrasound systems measure the propagation speed of the shear waves and convert it to Young’s modulus^1,9^, amplifying the difference and potential bias between the manual and the algorithm’s analysis, due to potentially differing formulae^5,8^. Since an additional conversion from Young’s modulus to shear modulus is required for accurate interpretation in muscle tissue^2,9^, further potential for bias is introduced as the formula used to calculate this relationship is based on approximations^8^. Therefore, previous work encouraged researchers to report SWE measurements in terms of SWV rather than mechanical moduli^5,8^, particularly discouraging the use of Young’s modulus, as it violates assumptions regarding tissue behavior even more than the shear modulus^2,9^. This is supported by the present results since the bias was smaller, and the overall agreement was higher in the analysis using SWV. We therefore aim to include the conversion to SWV directly within the software tool in a later release. However, this approach introduces the rounding error into the results of the algorithm as well, which needs to be addressed as a limitation of our software tool.

The present work has several limitations that need to be addressed. First, only clips of one muscle were used for the analysis. Although unlikely, the algorithm may face further challenges in muscles that are more difficult to scan. Second, the data used for the analyses did not cover the full range of elasticity values that the ultrasound device is technically capable of. Most measured values lie within a subset of possible numeric values. Therefore, we exported the data in an adapted elasticity range to increase the range of colors and therewith the contrasts. Using this additional manual step appeared necessary to achieve good measurements. Third, in Bland–Altman analyses normality of the data is assumed. Since not all variables, including the differences between the measurements in SWV, were normally distributed this might limit the results. However, the analyses were performed with raw and log-transformed data, and thereby covering most of the problems of non-normality.

When using the here presented algorithm for further research studies, the following limitations of the software tool need to be considered. The tool was developed to analyze SWE clips in DICOM format from Aixplorer ultrasound systems (SuperSonic Imagine, Aix-en-provence, France) and validated for Aixplorer clips. The automatic elastogram detection requires vertical image orientation, meaning the image containing the superimposed elastogram has to be displayed on the top, and the b-mode image on the bottom. Further, it is necessary to export the data as clips, as only clips, to our knowledge, display the orange frame around the elastogram, that is used for detection. If data is formatted differently, the image needs to be segmented manually, about which the user is instructed through MATLAB’s command window. The conversion of the elasticity values in the algorithm is based on the Aixplorer’s default colormap, ranging from blue (representing low values) to red (representing high values), displayed with 50% opacity, as this is the default setting and yielded the best results in the original algorithm^10^. Different opacity settings, will influence the performance of the tool and results are not validated. For optimal performance, we advise users to adjust the elasticity range prior to export of the clips to utilize the full color spectrum since the agreement of the automatic with the manual evaluation is best for the mid-scale color range. We have tested our tool with clips from two different Aixplorer generations (Ultimate V12 and Mach30). Although the software tool worked with both generations, we encourage users to test the tool in a small sample before trusting in the analysis. In the currently provided version of the algorithm values are expressed as Young’s modulus and must be recalculated to SWV or shear modulus to validly report the stiffness of muscle tissue. Lastly, the use of MATLAB limits the generalizability of the tool, as MATLAB is not an open-source option. In the future an executable version or a translation into an open source software such as python would be beneficial for the scientific community to overcome the dependence of MATLAB.

The present work aimed to validate the results of the software tool against the results obtained from the same measurement zone using the system provided ‘Q-Box tool’, averaged across all frames of an imaging sequence. Although the presented software tool enables the usage of large and temporally averaged measurement zones, it cannot answer the question whether this approach is superior to small measurement zones or not. Future research could target this question using the presented software tool and compare its results to simple small ROI manual analyses. Further, the presented software tool cannot account for insufficient data due to operator error during data acquisition.

## Conclusion

In conclusion, the proposed algorithm provides a valid method for analyzing SWE clips of muscle tissue from Aixplorer ultrasound systems. Although there is a significant, proportional bias within the analyses of the algorithm compared to manual analyses, this bias lies within the measurement error of SWE itself and is therefore clinically negligible. The algorithm can be used interchangeably with manual analyses, if values are expressed as SWV or logarithmic scaling is used. This algorithm can further enhance the quality of SWE measurements in musculoskeletal research by allowing researchers to conduct a standardized, semi-automated analysis of clips rather than single images in a time-efficient manner. Furthermore, no programming expertise is required to use the developed software making the tool more accessible and further streamlining the analysis process.

## Electronic supplementary material

Below is the link to the electronic supplementary material.

Supplementary Material 1

## Data Availability

All data generated or analyzed during this study are included in this published article and its supplementary information files. Information such as the algorithm and GUI can be obtained from GitHub [https://github.com/KatrinSkerl/EvaluateMuscleTissueWithElastography/tree/main].
