# Supplementary material for: Development and validation of a semi-automated algorithm to analyze shear wave elastography clips in muscle tissue
Source: Sci Rep. 2025 Jun 20;15:20147. doi: 10.1038/s41598-025-05154-2 (PMC12181364; doi:10.1038/s41598-025-05154-2)
Supplement: Supplementary file 1 — Supplementary Material 1 [file 41598_2025_5154_MOESM1_ESM.zip › SupplemetaryMaterial/Haueise_SupplementaryMaterial_final.pdf]

Supplementary Material

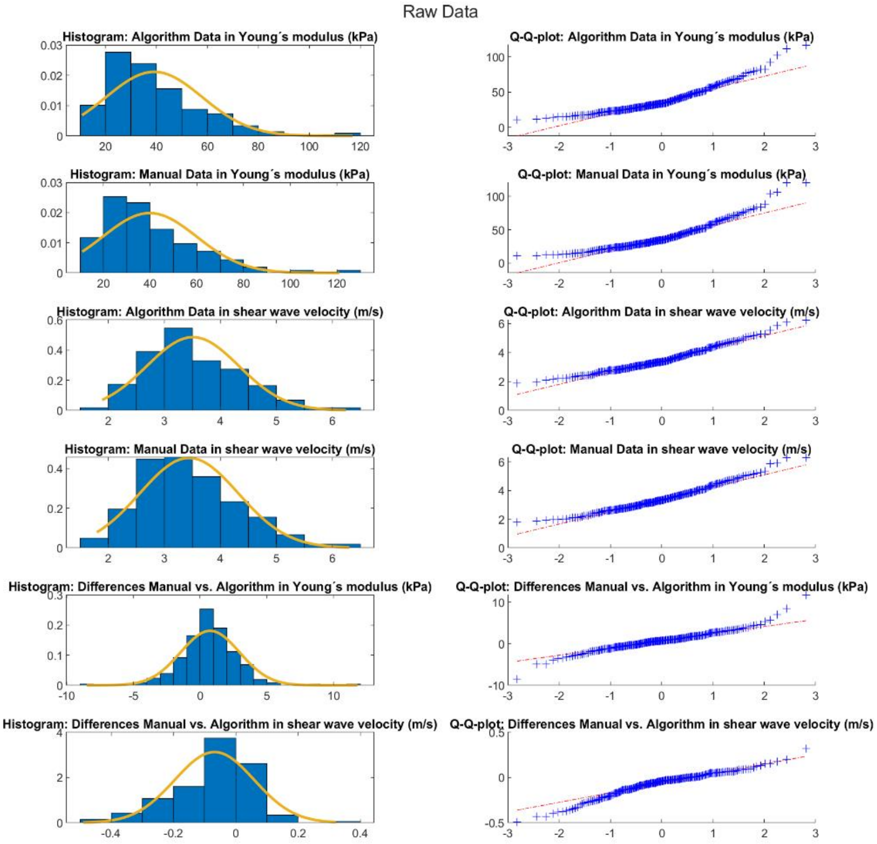

Figure S1: side-by-side histograms with probability density function curves of a normal distribution (yellow lines) and Q-Q-plots of algorithm data in Young’s modulus and SWV, manually analyzed data in Young’s modulus and SWV, and the differences between algorithm and manual measurements in Young's modulus and SWV.

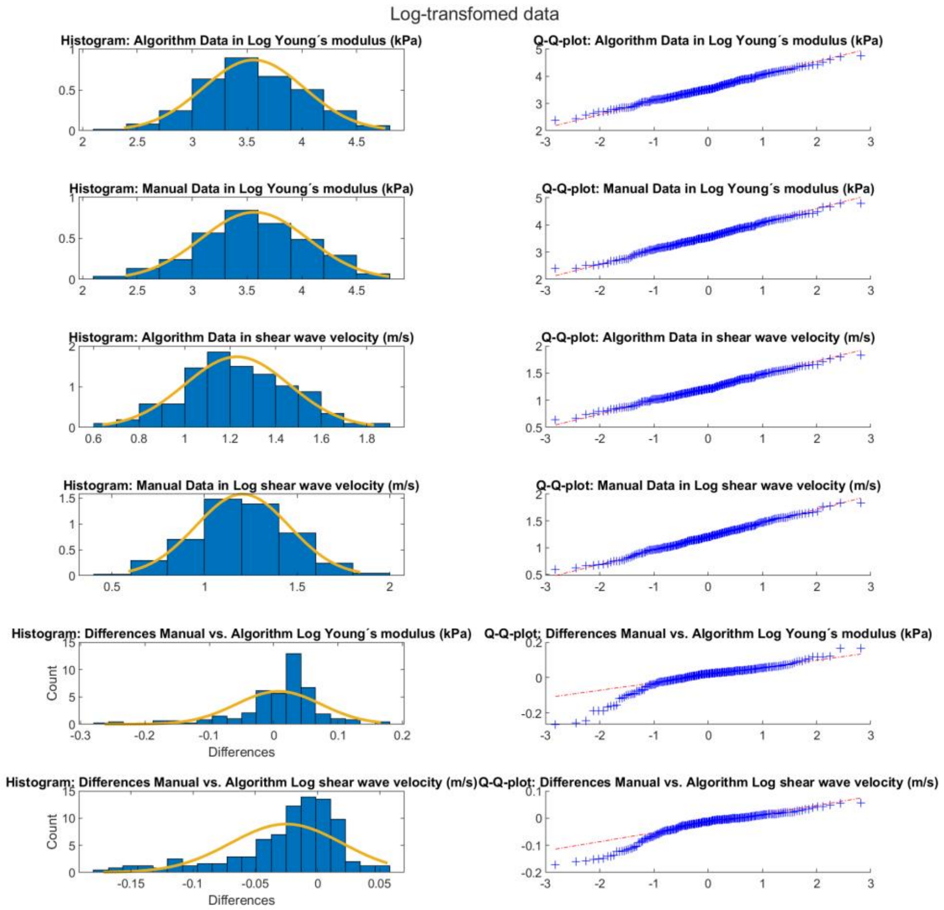

Figure S2: side-by-side histograms with probability density function curves of a normal distribution (yellow lines) and Q-Q-plots of log-transformed algorithm data in Young's modulus and SWV, log-transformed manually analyzed data in Young's modulus and SWV, and the differences between algorithm and manual measurements in log-transformed Young's modulus and log-transformed SWV.

The .xlsx-file 'Data_Algorithm_QBox.xlsx' contains the data used for this paper and is structured as follows:

- Column 1: filenames of the original raw data
- Column 2-3: mean and standard deviation of the algorithm's analysis, expressed as Young's modulus
- Column 4-5: mean and standard deviation of the algorithm's analysis, expressed as shear wave velocity
- Column 6-7: mean and standard deviation of the manual analysis, expressed as Young's modulus
- Column 8-9: mean and standard deviation of the manual analysis, expressed as shear wave velocity
- Column 10: log-transformed from the algorithm's analysis in Young's modulus
- Column 11: log-transformed from the manual analysis in Young's modulus
- Column 12: log-transformed from the algorithm's analysis in shear wave velocity
- Column 13: log-transformed from the manual analysis in shear wave velocity
